# Mediation of the malignant biological characteristics of gastric cancer cells by the methylated CpG islands in RNF180 DNA promoter

**DOI:** 10.18632/oncotarget.9494

**Published:** 2016-05-20

**Authors:** Jingyu Deng, Jiangtao Guo, Xiaofan Guo, Yachao Hou, Xingming Xie, Changyu Sun, Rupeng Zhang, Xiaohua Yu, Han Liang

**Affiliations:** ^1^ Department of Gastroenterology, Tianjin Medical University Cancer Hospital, City Key Laboratory of Tianjin Cancer Center and National Clinical Research Center for Cancer, Tianjin, China; ^2^ Department of Technical Quality, Tianjin Zhongxin Pharmaceutical Group Corporation Limited, Traditional Chinese Medicine Factory, Tianjin, China

**Keywords:** ring finger protein 180, methylation, proliferation, invasion, apoptosis

## Abstract

We previously demonstrated that the methylation of ring finger protein 180 (RNF180) DNA promoter was specific to gastric cancer tissues. We reported that four hypermethylated CpG islands, namely, CpG-116, CpG-80, CpG+97, and CpG+102, in RNF180 promoter were significantly associated with the postoperative overall survival of gastric cancer patients. Correlation analysis revealed that the methylated status of CpG islands was significantly associated with the lymph node metastasis of gastric cancer. We formulated four types of MGC-803 cells with the specific demethylation of one of the four CpG islands through vector transfection method. Conventional detections for the biological characteristics of cancer cells showed that 1) the methylation of CpG+102 island in RNF180 DNA promoter could remarkably influence the comprehensively malignant biological characteristics of gastric cancer cells, including their proliferation, invasion, cell cycle, anti-apoptosis, and tumorigenicity. 2) The CpG+97 island, in addition to the CpG+102 island, should be considered as the other key methylated locus in RNF180 DNA promoter to mediate the malignant biological characteristics of gastric cancer cells. The methylated status of the key CpG islands of RNF180 DNA promoter may be used to predict the variations of the malignant biological characteristics of gastric cancer cells. The proposed method is a promising molecular therapy for gastric cancer.

## INTRODUCTION

DNA methylation, which is the main epigenetic feature of DNA, mainly functions in gene transcriptional regulation and activates many cellular processes, including oncogenesis [1]. Thus far, various human malignancies are characterized by aberrancies in DNA methylation [2]. CpG islands are CpG-rich regions located in more than half of the promoters of mammalian genes; these islands exhibit exceptional and global unmethylated patterns [3–5]. The methylation of CpG islands modifies the transcriptional activity of key proliferation genes or transcription factors involved in cell growth suppression or promotion [6]. Gene-specific hypermethylation at certain tumor-suppressor gene sites and transcriptional inactivation by cytosine methylation at promoter CpG islands may silence tumor suppressor genes in oncogenesis [7, 8]. In several human cancer types, subgroups defined by distinctive methylation patterns have been linked to several features, such as tumor size in breast cancer [9], tumor type in lung [10], and tumor histology in glioma [11]. First proposed in 1999 by Toyota [12], the CpG island methylator phenotype (CIMP) in colorectal cancer is a well-studied methylation-defined subgroup. CIMP is defined as a widespread and increased level of DNA methylation in various human malignancies; it also represents a subclass of tumors with distinctive clinicopathological and molecular features[13]. However, a screen of methylated genes that can represent distinctive characteristics from various gastric tumors is difficult to accomplish because of the heterogeneity of gastric cancer tissues. The function of specially methylated CpG islands in DNA promoters in gastric cancer has been extensively investigated. In a previous study, the methylation of ring finger protein 180 (RNF180) DNA promoter is specific to gastric cancer tissues, and four hypermethylated CpG islands, namely, CpG-116, CpG-80, CpG+97, and CpG+102, in RNF180 promoter are significantly associated with the postoperative overall survival of gastric cancer patients [14]. Correlation analyses revealed that the methylated status of CpG islands is significantly associated with the lymph node metastasis of gastric cancer [14]. Therefore, various methylated CpG islands may elicit different effects on the mediation of the biological behaviors of gastric cancer cells during canceration. This present study aimed to investigate whether CpG-116, CpG-80, CpG+97, and CpG+102 in RNF180 DNA promoter can moderate the malignant biological characteristics of gastric cancer cells to alter the progression of this disease.

## RESULTS

### Detection of the CpG island demethylation of RNF180 DNA promoters in various MGC-803 cell lines

**Figure 1:**
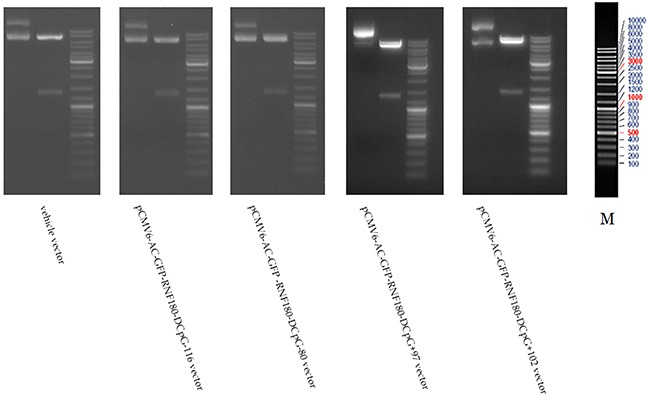
Macrorestriction maps for MGC-803 cells transfected with various vectors

**Figure 2:**
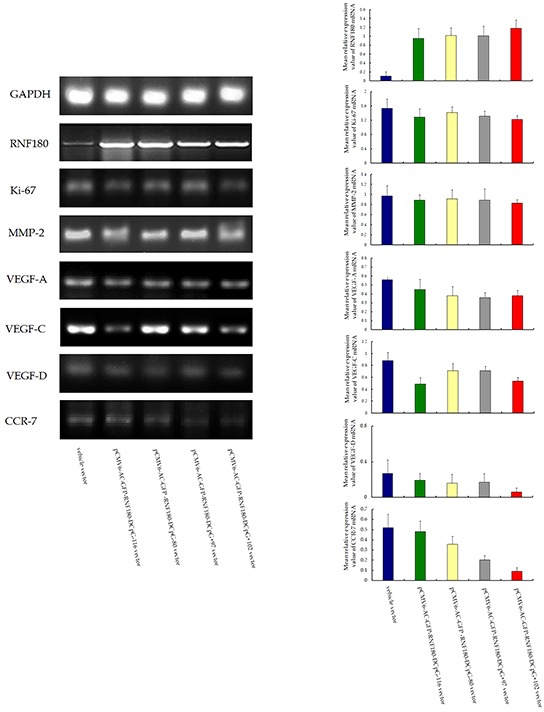
Bisulphite sequencing figures for MGC-803 cells transfected with various vectors

**Figure 3:**
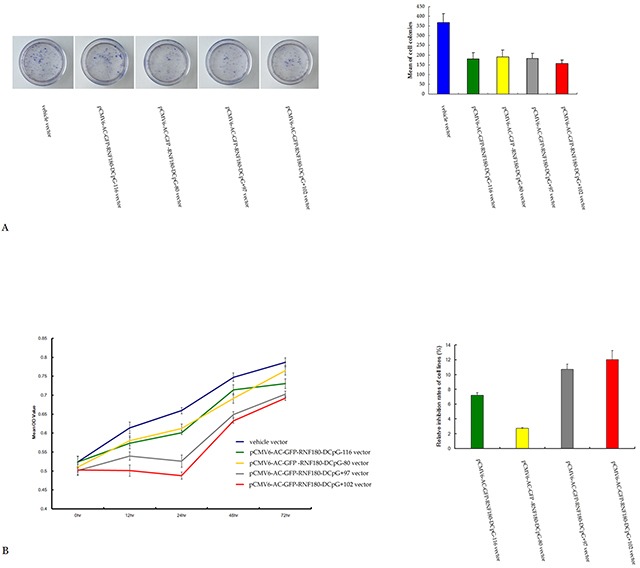
RT-PCR detection RNF180mRNA expression for MGC-803 cells transfected with various vectors

Figure 1 shows that the four types of RNF180 DNA promoter fragments, including the various cytosine-thymine conversion in corresponding CpG islands (CpG-116, CpG-80, CpG+97, or CpG+102), were successfully subcloned in the pCMV6-AC-GFP-RNF180 vectors. With BGS detection, we demonstrated that the four cancer cell lines transfected with the various demethylated CpG island vectors were manufactured (Figure 2). Subsequently, we also detected the transcriptional levels (mRNA) of RNF180 gene in four kinds of MGC-803 cell lines, which were transfected with the various demethylated CpG island vectors; and MGC-803 cell line, which was transfected with the vehicle vector. As expected, all four kinds of MGC-803 cell lines transfected with various demethylated CpG island vectors (pCMV6-RNF180-DCpG-116, pCMV6-RNF180-DCpG-80, pCMV6-RNF180-DCpG+97, and pCMV6-RNF180-DCpG+102) showed considerably increased RNF180 mRNA mean relative expression values (MREV) (MREV_CpG-116_ =0.862, MREV_CpG-80_ =0.946, MREV_CpG+97_ =1.011, and MREV_CpG+102_ =1.007). In comparison, MGC-803 cell line transfected with vehicle vector revealed the approximate silence of RNF180 mRNA (MREVvehicle=0.099) (P_CpG-116 VS vehicle_ <0.001, P_CpG-80 VS vehicle_ =0.001, P_CpG+97 VS vehicle_ <0.001, and P_CpG+102 VS vehicle_ <0.001) (Figure 3). Therefore, we were convinced that the four kinds of MGC-803 cell lines transfected with various demethylated CpG island vectors may serve as potential key sites of re-expressing RNF180 for the regulation the biological functions of MGC-803 cells.

### CpG island demethylation of RNF180 DNA promoter activates gene transcriptions of malignant biological characteristics of MGC-803 cells

The potential molecular mechanism of CpG island demethylation in the RNF180 DNA promoter regulation was also explored. The malignant biological characteristics of gastric cancer cells, or the MREV of the common genes involved in the tumor progression, were used to assess the expression differences among the four MGC-803 cell lines transfected with various demethylated CpG island vectors (pCMV6-RNF180-DCpG-116, pCMV6-RNF180-DCpG-80, pCMV6-RNF180-DCpG+97, and pCMV6-RNF180-DCpG+102) and MGC-803-vehicle cell line. With the RT-PCR assay, we demonstrated that all four kinds of MGC-803 cell lines transfected respectively with various demethylated CpG island vectors presented lower MREVs of Ki-67, MMP-2, VEGF-A, VEGF-C, and VEGF-D than those of the MGC-803-vehicle cell line. Notably, MGC-803-pCMV6-RNF180-DCpG+102 cancer cell lines presented the lowest MREV of Ki-67 (P_CpG+102 VS vehicle_ =0.031), MMP-2 (P_CpG+102 VS vehicle_ =0.034), VEGF-A (P_CpG+102 VS vehicle_ =0.030), VEGF-D (P_CpG+102 VS vehicle_ =0.118), and CCR-7 (P_CpG+102 VS vehicle_ =0.001) among the above-mentioned cancer cell lines. Additionally, MGC-803-pCMV6-RNF180-DCpG+102 cancer cell lines presented the second lowest MREV of VEGF-C (P_CpG+102 VS vehicle_ =0.045) among the above-mentioned cancer cell lines, following by MGC-803-pCMV6-RNF180-DCpG-116 cancer cell line (P_CpG+97 VS vehicle_ =0.036). These gene transcription detection results suggested that the methylation of CpG+102 island of RNF180 promoter may enhance the malignant biological characteristics of MGC-803 cells and contribute to the progression of gastric cancer, including lymphatic invasion.

### CpG island demethylation of RNF180 DNA promoter inhibits MGC-803 cell proliferation and viability

**Figure 4:**
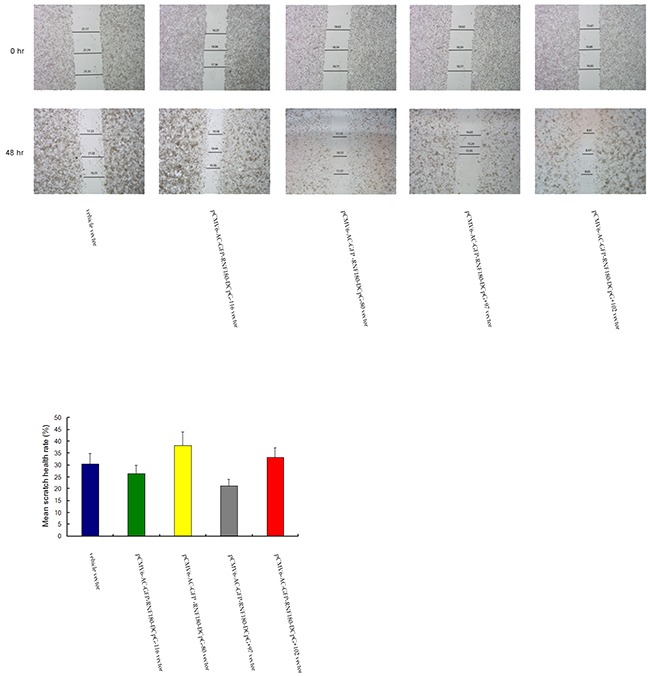
Colony formation **A.** and MTT assay **B.** for MGC-803 cells transfected with various vectors.

We detected the effect of various CpG island demethylation of RNF180 DNA promoter on MGC-803 cell growth and viability using colony formation and MTT assay. The colonies formed by MGC-803-vehicle cell line were significantly more than those of the MGC-803 cell lines transfected with various demethylated CpG island vectors (vehicle 368±47, pCMV6-RNF180-DCpG-116 182±31, pCMV6-RNF180-DCpG-80 192±34, pCMV6-RNF180-DCpG+97 183±26, pCMV6-RNF180-DCpG+102 158±18; P_CpG-116 VS vehicle_ =0.017, P_CpG-80 VS vehicle_ =0.026, P_CpG+97 VS vehicle_ =0.021, and P_CpG+102 VS vehicle_ =0.005) (Figure 4A). MGC-803-pCMV6-RNF180-DCpG+102 cancer cell line was detected to present the highest mean 72 h inhibition rate (IR) of 12.07% in all cell lines. The mean 72 h IRs of the three other cancer cell lines transfected with the demethylated CpG island vectors (pCMV6-RNF180-DCpG-116, pCMV6-RNF180-DCpG-80, and pCMV6-RNF180-DCpG+97) were 7.22%, 2.75%, and 10.73%, respectively (Figure 4B). The colony formation and MTT assay results suggested that the methylation of CpG islands of RNF180 promoter was capable of enhancing the proliferation and viability of MGC-803 cancer cells. However, the methylation of the CpG+102 island of RNF180 DNA promoter could remarkably enhance the proliferation and viability of MGC-803 cells.

### CpG island demethylation of RNF180 DNA promoter inhibits MGC-803 cell migration

**Figure 5:**
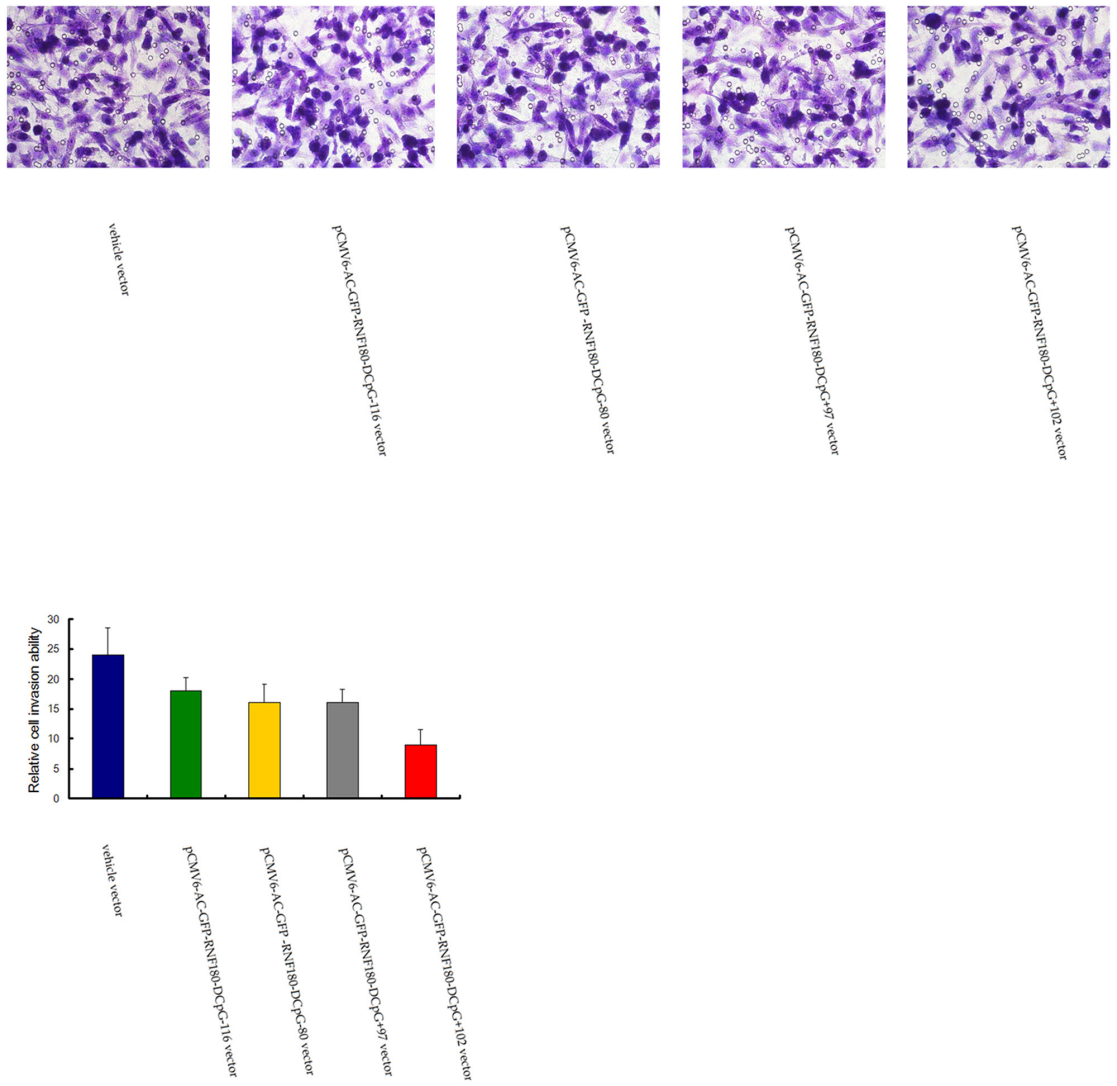
Cell-based scratch assay for MGC-803 cells transfected with various vectors

Scratch assays were performed to observe the function of various CpG island demethylation of RNF180 DNA promoter in MGC-803 cell migration. As shown in Figure 5, cell migration was significantly inhibited in the two MGC-803 cell lines transfected with demethylated CpG island vectors (pCMV6-RNF180-DCpG-116 and pCMV6-RNF180-DCpG+97) compared with that in the MGC-803 cell line transfected with vehicle vector. The scratch health rates of all five cell lines were 30.41%, 26.25%, 38.17%, 21.25%, and 33.26% for MGC-803-vehicle, MGC-803-pCMV6-RNF180-DCpG-116 cells, MGC-803-pCMV6-RNF180-DCpG-80 cells, MGC-803-pCMV6-RNF180-DCpG+97 cells, and MGC-803-pCMV6-RNF180-DCpG+102 cells at 48 h time-point, respectively. This finding indicated that methylation of CpG-116 and CpG+97 islands of RNF180 DNA promoter could significantly enhance the migration of gastric cancer cells (P_CpG-116 VS vehicle_ =0.031 and P_CpG+97 VS vehicle_ =0.024).

### CpG island demethylation of RNF180 DNA promoter inhibits MGC-803 cell invasion

**Figure 6:**
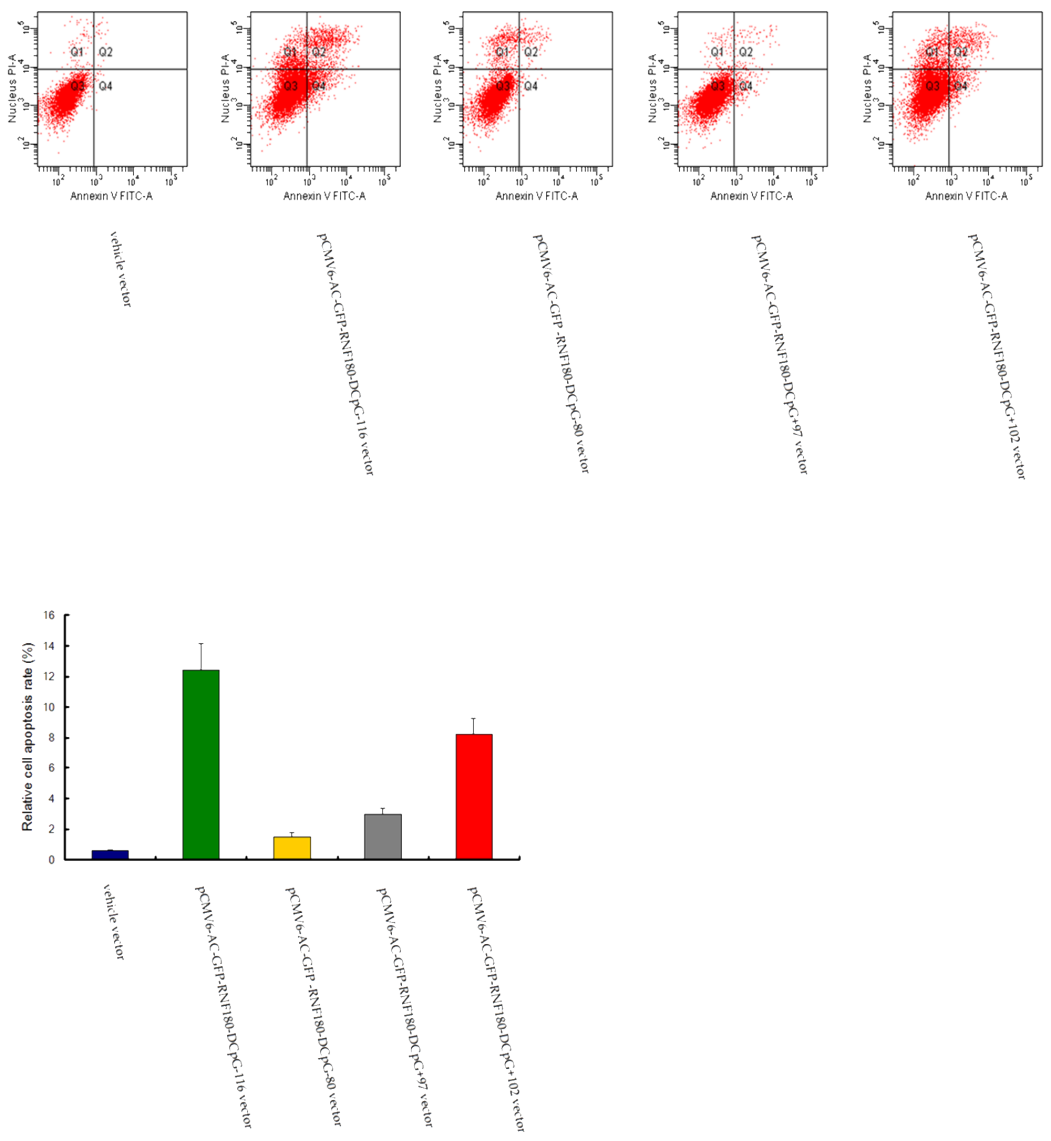
Transwell tumor cell invasive assay for MGC-803 cells transfected with various vectors

The various methylated CpG islands of RNF180 promoter on the invasiveness of MGC-803 cells were studied using Matrigel model. We found that the number of invaded cells was significantly lower in the four kinds of MGC-803 cell lines transfected with the demethylated CpG island vectors (pCMV6-RNF180-DCpG-116, pCMV6-RNF180-DCpG-80, pCMV6-RNF180-DCpG+97, and pCMV6-RNF180-DCpG+102) compared with those in the MGC-803 cell line transfected with vehicle vector (Figure 6). The mean cell number of all five cell lines were 24, 18, 16, 16, and 9 for MGC-803-vehicle, MGC-803-pCMV6-RNF180-DCpG-116 cells, MGC-803-pCMV6-RNF180-DCpG-80 cells, MGC-803-pCMV6-RNF180-DCpG+97 cells, and MGC-803-pCMV6-RNF180-DCpG+102 cells at 24 h time-point respectively. This result indicated that the methylation of four CpG islands (CpG-116, CpG-80, CpG+97, and CpG+102) of the RNF180 DNA promoter could enhance the invasion of gastric cancer cells (P_CpG-116 VS vehicle_ =0.041, P_CpG-80 VS vehicle_ =0.020, P_CpG+97 VS vehicle_ =0.023, and P_CpG+102 VS vehicle_ <0.001). Of the four above-mentioned CpG islands, the methylation of the CpG+102 island of RNF180 DNA promoter remarkably enhanced the invasion of MGC-803 cells.

### CpG island demethylation of RNF180 DNA promoter improves MGC-803 cell apoptosis

**Figure 7:**
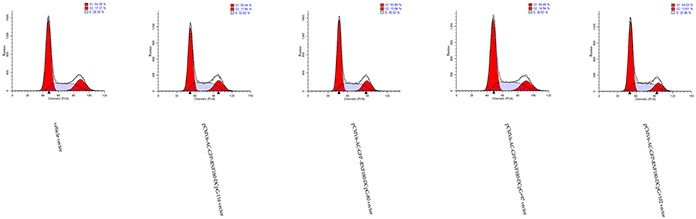
Cell apoptosis detection for MGC-803 cells transfected with various vectors

Compared with the cell apoptosis in the MGC-803 cell line transfected with vehicle vector, cell apoptosis rates were significantly enhanced in the four MGC-803 cell lines transfected with the demethylated CpG island vectors (pCMV6-RNF180-DCpG-116, pCMV6-RNF180-DCpG-80, pCMV6-RNF180-DCpG+97, and pCMV6-RNF180-DCpG+102) (Figure 7). The mean cell apoptosis rates of all five cell lines were 0.6%, 12.4%, 1.5%, 3.0%, and 8.2% for MGC-803-vehicle, MGC-803-pCMV6-RNF180-DCpG-116 cells, MGC-803-pCMV6-RNF180-DCpG-80 cells, MGC-803-pCMV6-RNF180-DCpG+97 cells, and MGC-803-pCMV6-RNF180-DCpG+102 cells respectively. This finding indicated that the methylation of four CpG islands (CpG-116, CpG-80, CpG+97 and CpG+102) of RNF180 DNA promoter could enhance the anti-apoptosis of MGC-803 cells (P_CpG-116 VS vehicle_ <0.001, P_CpG-80 VS vehicle_ =0.036, P_CpG+97 VS vehicle_ =0.011, and P_CpG+102 VS vehicle_ <0.001). Of the four abovementioned CpG islands, the methylation of CpG-116 island of RNF180 DNA promoter could remarkably enhance the invasion of MGC-803 cells, followed by the methylation of CpG+102 island.

### CpG island demethylation of RNF180 DNA promoter inhibits MGC-803 cell cycle in G2 phase

**Figure 8:**
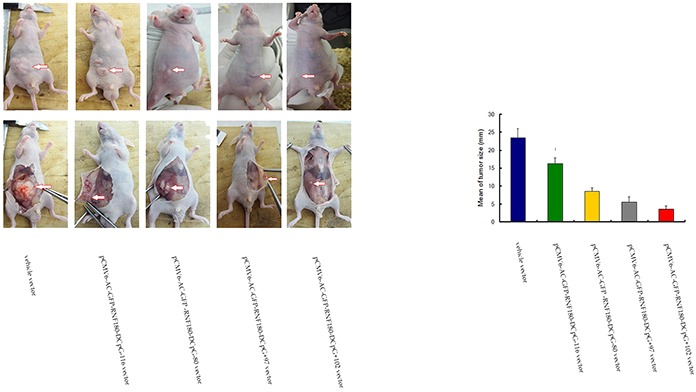
Cell cycle assay for MGC-803 cells transfected with various vectors

Flow cytometry was adopted to detect the cell cycle changes of MGC-803 gastric cells stimulated by the demethylation of various CpG islands in RNF180 DNA promoter. MGC-803-pCMV6-RNF180-DCpG+97 and MGC-803-pCMV6-RNF180-DCpG+102 cell lines presented higher cell counts in the S phase and lower cell counts in the G2 phase compared with those in the MGC-803-vehicle at a 72 h time-point. The mean diploid ratios of G2 phase cell counts of three cancer cell lines transfected with the demethylated CpG islands (CpG+97, CpG+102, and vehicle) were 14.94%, 13.01%, and 17.27%, respectively (Figure 8). The flow cytometry assay results suggested that the methylation of CpG islands (CpG+97 and CpG+102) of the RNF180 promoter could enhance cell synthesis and improve the mitosis of MGC-803 cancer cells.

### CpG island demethylation of RNF180 DNA promoter inhibits tumor growth in nude mice

**Figure 9:**
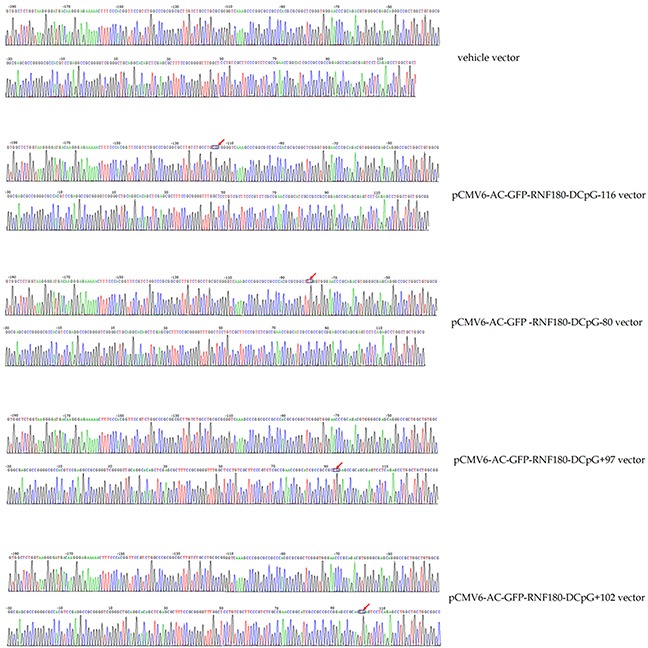
Tumor size measuring for MGC-803 cells transfected with various vectors

We explored whether the CpG island demethylation of RNF180 DNA promoter could inhibit the growth of GC cells in nude mice in vivo. The mean tumor sizes in nude mice transfected with the four kinds of MGC-803 cell lines with various CpG island demethylation in RNF180 DNA promoter were significantly smaller than those transfected with MGC-803-vehicle cells at a four-week time-point after cell implantation (P_CpG-116 VS vehicle_ =0.031, P_CpG-80 VS vehicle_ =0.014, P_CpG+97 VS vehicle_ =0.001, and P_CpG+102 VS vehicle_ <0.001). This finding indicates that the methylation of four CpG islands (CpG-116, CpG-80, CpG+97, and CpG+102) of RNF180 DNA promoter could improve the tumorigenicity of MGC-803 cells (Figure 9). Of the four CpG islands previously mentioned, the methylation of CpG+102 island of RNF180 DNA promoter could considerably enhance the tumorigenicity of MGC-803 cells, followed by the methylation of CpG+97 island.

## DISCUSSION

The ubiquitin-proteasome system (UPS) maintains the dynamic equilibrium of the abundance and activity of various cellular proteins; UPS is significantly associated with oncogenesis and tumor progression [15, 16]. RNF180 is an E3 ubiquitin ligase; it is implicated in the ubiquitin-proteasome pathway supported by its binding to the UbcH6 ubiquitin-conjugating enzyme and by its trans-ubiquitination-enhancing activities [17]. RNF180 participates in tumorigenesis and likely suppresses the progression of human cancers [18, 19]. The loss or downregulation of RNF180 is associated with a significantly increased risk of cancer-related deaths of gastric cancer patients [19]. Furthermore, the promoter methylation of RNF180 DNA, which directly mediates RNF180 transcription silencing, may alter the malignant biological characteristics of gastric cancer cells to accelerate gastric cancer progression [19]. The methylation of various CpG islands in the RNF180 DNA promoter may elicit different effects on lymph node metastasis associated with gastric cancer [14, 20]. CpG-116, CpG-80, CpG+97, and CpG+102 in RNF180 promoter are negatively related to the overall survival of gastric cancer patients, as revealed by multivariate analysis [14]. This finding provided a basis for investigations on the methylated status of different CpG islands in RNF180 DNA promoters to elucidate the detailed mechanism of RNF180 suppression in gastric cancer progression.

In this study, four kinds of RNF180 DNA promoter fragments with demethylated CpG-116, CpG-80, CpG+97, and CpG+102 were designed by using cytosine-thymine conversions in the locus of RNF180 promoter. Subsequently, four MGC-803 cancer cell lines were transfected with the four RNF180 DNA promoter fragments with demethylated CpG islands. The transcriptional levels of RNF180 (mRNA) in the four cancer cell lines considerably increased compared with those in the MGC-803-vehicle cell line. Therefore, the demethylation of CpG-116, CpG-80, CpG+97, and CpG+102 could increase the transcriptional level of RNF180 in gastric cancer cells. This finding could demonstrate that the methylation of the four CpG islands of the DNA promoter might reveal the potential inhibition, transcription, expression, and biological function of RNF180 gene. These conclusions provided a solid basis for further studies on the biological effects of the methylation of different CpG islands in RNF180 DNA promoter on gastric cancer cell. These conclusions could help elucidate the molecular mechanisms by which RNF180 mediates gastric cancer progression.

We applied RT-PCR to detect the mRNA levels of the common genes related to the malignant biological characteristics of gastric cancer cells and to explore the differences in biological characteristics among five MGC-803 cell lines. RT-PCR assay results revealed that the demethylation of CpG-116, CpG-80, CpG+97, and CpG+102 in RNF180 DNA promoter could partially suppress the transcriptional levels of common genes related to the malignant biological characteristics of gastric cancer cells, including proliferation, invasion, angiogenesis, lymphangiogenesis, and chemotaxis.

The demethylation of CpG+102 island in RNF180 DNA promoter could significantly increase the transcriptional value of RNF180 (mRNA) in the MGC-803 cell line. Furthermore, the demethylation of CpG+102 island in the RNF180 DNA promoter evidently suppressed the transcriptions of the genes related to the malignant biological characteristics of MGC-803 cells. This result indicated that the methylated status of the CpG+102 island in the RNF180 DNA promoter might be the intensive site associated with gastric cancer progression among four CpG islands in RNF180 DNA promoter. The methylation of the CpG+102 island in RNF180 DNA promoter could promote tumor progression by regulating the malignant biological abilities of MGC-803 cells, including proliferation, invasion, cell cycle, and anti-apoptosis, and by promoting the tumorigenicity of MGC-803 cells in vivo. Therefore, the methylated status of the CpG+102 island in RNF180 DNA promoter could be a promising molecular therapy to inhibit the malignant characteristics of gastric cancer cells.

Similar to the demethylation of the CpG+102 island, the demethylation of the CpG+97 island in the RNF180 DNA promoter could significantly increase the transcriptional level of RNF180 (mRNA) in the MGC-803 cell line. MTT assay and tumor growth in vivo experiment demonstrated that CpG+97 island was another intensive locus that evidently promoted the tumorigenicity of MGC-803 cells. Furthermore, the methylation of CpG+97 island in RNF180 DNA promoter significantly contributed to the anti-apoptosis of cancer cells by changing the cell cycle and enhancing the migration capability of gastric cancer cells. The significant increase in the CCR-7 transcriptional level in the MGC-803-pCMV6-RNF180-DCpG+97 cell line (following the MGC-803-pCMV6-RNF180-DCpG+102 cell line) indicated that the CpG+97 island in RNF180 DNA promoter was the other potential trigger point that promoted the chemotactic movement in the migration and invasion of gastric cancer cells. Lastly, the methylation of CpG+97 island in the RNF180 DNA promoter was associated with the invasive ability of MGC-803 cells through Trans-well chambers. This finding indicated that the methylated status of CpG+97 island in RNF180 DNA promoter was an important contributor to tumor spread. In addition to the methylated status of CpG+102 island, the methylated status of CpG+97 island could be considered as another important locus in the RNF180 DNA promoter to predict the malignancy of gastric cancer cells. The methylated status of CpG-116 and CpG-80 islands in the RNF180 DNA promoter mediate several malignant biological behaviors of MGC-803 cells. Therefore, these islands were not competent to comprehensively reflect the biological effects of RNF180 promoter methylation on gastric cancer cells.

We proposed to investigate various CpG islands in the RNF180 DNA promoter to reveal the key locus of the methylated fragment in the promoter, which could potentially mediate the malignant biological behavior of gastric cancer cells, in a manner similar to the definition of CIMP in tumors. On the basis of our previous studies, we demonstrated that the methylation of CpG-116, CpG-80, CpG+97, and CpG+102 in the RNF180 DNA promoter was significantly associated with the prognosis of gastric cancer patients. However, we should elucidate the most intensively methylated CpG islands that induce the progression of gastric cancer in the RNF180 DNA promoter to develop precise molecular treatments for gastric cancer. The methylated status of the CpG+102 island should be considered the first preferred locus in the RNF180 DNA promoter for the accurate prediction of malignant biological behaviors of gastric cancer cells. This method could be a promising molecular therapy for gastric cancer. The methylated status of the CpG+97 island might also be an alternative to the methylated status of the CpG+102 island in the RNF180 DNA promoter to predict the progression of gastric cancer.

## MATERIALS AND METHODS

### Cell lines

Gastric cancer cell line (MGC-803) was purchased from the Type Culture Collection of the Chinese Academy of Sciences, (Shanghai, China). Cells were cultured in RPMI-1640 medium (Thermo Electron Corporation, Beijing, China) supplemented with 10% fetal bovine serum (Life Tech, Mulgrave Vic, Australia) and incubated in 5% CO2 at 37°C. The medium was changed twice a week.

### Semi-quantitative reverse transcription polymerase chain reaction (RT-PCR) analysis

Total RNA was extracted from cell lines by Trizol reagent (Invitrogen, Carlsbad, CA). The messenger RNA (mRNA) expression levels of the RNF180, Ki-67, matrix metalloproteinase 2 (MMP-2), vascular endothelial growth factor A/C/D (VEGF-A/C/D), and chemokine receptor 7 (CCR-7) were determined by semi-quantitative Reverse Transcription Polymerase Chain Reaction (RT-PCR) detection. The PCR Cycling conditions for all sequences were 40 cycles of denaturation at 94°C for 4 minutes, annealing at 94°C for 30 seconds, and extension at 58°C for 30 seconds followed by a final extension at 72°C for 10 minutes and 4°C for 1 minute. All PCR product electrophoreses were performed on a 2% agarose gel with ethidium bromide and visualized using the Gel Imager system (Asia Xingtai Mechanical and Electrical Equipment Company, Beijing, China). Mean gray value of relative expression of mRNA was defined as the ratio between the mean gray values of the given mRNA expression and the mean gray value of the GAPDH expression. Primers designed and utilized for detected genes were shown in Table 1.

**Table 1:** Primers utilized for target genes detection in all MGC-803 cancer cell lines

Genes	Primer sequences	Length
RNF180	5′-TCTGACTTTCCTGATGGACCTG/CCTGAGTATTTACCCTGCTTCTGT-3′	(175bp)
Ki-67	5′-GACGGCCACAAACTCCTAAA/TGCTCTTTCCATCTCCTGCT-3′	(264bp)
MMP-2	5′-AGCTCCCGGAAAAGATTGAT/TTTTGCTCCAGTTAAAGGCG-3′	(215bp)
VEGF-A	5′-CCTTGCCTTGCTGCTCTA/ATGTCCACCAGGGTCTCG-3′	(150bp)
VEGF-C	5′-GCCCCAAACCAGTAACAATCA/CAGCATCCGAGGAAAACATAAA-3′	(222bp)
VEGF-D	5′-CTGCCTGATGTCAACTGCTTAG/AGATGATCGCTTCACTGGTCC-3′	(295bp)
CCR-7	5′-TGTGGTCGTGGTCTTCATAG/GCGTACAAGAAAGGGTTGAC-3′	(180bp)
GAPDH	5′-TGGGTGTGAACCATGAGAAGT/TGAGTCCTTCCACGATACCAA-3′	(124bp)

### Construction vectors and transfection

The RNF180 vector was generated by PCR cloning with a mammalian expression vector (pCMV6-AC-GFP) (Origene, USA). The cDNA corresponding to the open reading frame of RNF180 transcript was obtained by RT-PCR amplification of MGC-803 gastric cancer cells’ RNA. Four kinds of RNF180 DNA promoter fragments, including the various cytosine - thymine conversion in corresponding CpG islands (CpG-116, CpG-80, CpG+97, or CpG+102), were subcloned in the pCMV6-AC-GFP-RNF180 vectors to meet requirements of manufacture the different demethylated CpG islands in RNF180 DNA promoter. The identification of all pCMV6-AC-GFP -RNF180-demethylated island vectors and vehicle vector was performed by using the restriction enzyme digestion method (shown in Figure 1). Subsequently, MGC-803 cell line (2×10^5^/well) was transfected with the above-mentioned four kinds of RNF180 DNA promoter vectors (including MGC-803-pCMV6-RNF180-DCpG −116, MGC-803-pCMV6-RNF180-DCpG-80, MGC-803-pCMV6-RNF180-DCpG+97, or MGC-803-pCMV6-RNF180-DCpG+102) and vehicle vector (MGC-803-vehicle) using LipofectamineTM 2000 transfection reagent (Invitrogen, NewYork, USA). Transfected cells were selected with Opti-MEM (Invitrogen, NewYork, USA) for 48 hours (hr). Colonies were fixed with methanol/acetone (1:1), stained with Trypan Blue, and counted [19].

### DNA extraction and sodium bisulfite treatment

Genomic DNA was extracted from gastric cancer cells using QIAamp DNA mini kit (Qiagen, Valencia, CA) following the manufacturer's instructions. Sodium bisulphite modification of genomic DNA was performed by using the EZ DNA Methylation-GoldTM Kit (Zymo Research, Hornby, Canada).

### Bisulphite genomic sequencing

Cells were detected the quantitatively methylated analysis of RNF180 promoter with the bisulphite genomic sequencing method (BGS). Hot start PCR with the bisulfite-treated DNA was performed with a 318bp PCR product spanning promoter region from −192bp to 126bp relative to the transcription start site of RNF180. Forty-three CpG islands were identified to be contained in the promoter region of RNF180. The sequences of PCR primers were as follows: F:5′-GTGGTTTTGGTAAGGGGATGAT-3′; R:5′-CCAACAACCAAACTCTAAAAA CTC-3′. The purified PCR products were cloned into the pUC18-T vector (Biodee, Beijing, China), and six clones for each sample were randomly selected and sequenced by Shanghai Sangon Co.(Shanghai, China).

### Colony formation assay

Cells were selected with G418 sulfate (Merck, Darmstadt, Germany) at 0.4 mg/mL for 2 weeks. Colonies were fixed with methanol/acetone (1:1), stained with gentian violet, and counted. Colonies with cell numbers of more than 50 cells per colony were counted. All experiments were performed in triplicate wells 3 times each.

### Cell viability analysis

Cells were seeded in each well of a 96-well plate 24 hr prior to treatment. Dimethylthiazolyl-2-5-diphenyltetrazolium bromide (MTT) dye solution (Sigma, St. Louis, MO, USA) was added into the 96-well plate 20 hr post treatment. The plate was incubated at 37°C for 4 hr, and the treatment terminated by adding stop solution (isopropanol with 0.04 N HCl). MTT was cleaved by live cells to a colored formazan product. The supernatant was discarded and 150 μL DMSO was added to each well, mixed evenly, and the absorbance (A) was measured at 450 nm within 10 min using a DG-5031 ELISA Reader [21].

### Cell-based scratch assay

All cell lines were cultured in a 6-well culture plate for 24 hr up to 90%-100% confluences of the base was filled. Scratched wound lines on the upside of cultured cells were created by 200 μL yellow micropipette tip. The scratched cells were washed with PBS after removal of culture media. The migration distance was imaged and measured at different time (cells cultured for 0, 24, 48hr) points using a microscope (Olympus, Tokyo, Japan) by Image J program (NIH, USA) [22].

### Transwell tumor cell invasion assay

Cell invasion was quantified in vitro using Transwell chambers with polycarbonate membrane filters (8-μm pore size) coated with a Matrigel™ (Sigma, St. Louis, USA). Briefly, 48 hours after transfection, cells were washed twice using DMEM and seeded in triplicate in the inner chamber of the insert containing 200 μl of serum-free medium. About 700 μl of medium containing 10% fetal bovine serum was added to the lower chamber. The plates were incubated for 24 hr at 37°C. Then, the non-invading cells from the interior of the inserts were gently removed using a cotton-tipped swab. The cells that had invaded into the bottom surface of the filter were fixed with methanol and stained with hematoxylin. The invasive ability was determined by counting the penetrating cells under a microscope at ×400 magnification on 5 random fields in each well [23].

### Apoptosis assay

The percentage of apoptotic cells was ascertained by dual staining of cells with Annexin V and propidium iodide [24]. All kinds of cells (2.5 × 10^5^/ml) were incubated with or without AG–4 (5.4 μM, 48 h) in presence or absence of various inhibitors at 37°C, 5% CO2. Cells were then washed twice in PBS and resuspended in Annexin V binding buffer (10 mM HEPES, 140 mM NaCl, 2.5 mM CaCl2; pH 7.4). Annexin V-FITC was then added according to the manufacturer's instructions and incubated for 15 minutes under dark conditions at 25°C. Propium iodide (0.1 μg/ml) was added just prior to acquisition. Data was acquired using a FACS Aria flow cytometer (Becton Dickinson) at an excitation wavelength of 488 nm and an emission wavelength of 530 nm and analyzed with BD FACS Diva software (Becton Dickinson).

### Cell cycle distribution analysis

For the determination of cell cycle phase distribution of nuclear DNA, all five kinds of cells (1×10^6^ cells) were harvested. Then, cells were fixed with 3% p-formaldehyde, permeabilized with 0.5% Triton X-100, and nuclear DNA was labeled with propidium iodide (125 μg/mL) after RNase treatment using Cycle Test Plus DNA reagent kit. Cell cycle phase distribution of nuclear DNA was determined on FACS Calibur using Cell Quest Software (Becton-Dickinson). Histogram display of DNA content (x-axis, fluorescence) versus counts (y-axis) has been displayed. Cell Quest statistics was employed to quantitate the data at different phases of the cell cycle.

### In vivo tumorigenicity

All five kinds of cells (1 × 10^6^ cells in 0.1 mL phosphatebuffered saline) were injected subcutaneously into the dorsal flank of 3-week-old male nude mice, separately. Tumor diameter was measured every 2 or 3 days until 4 weeks. Tumor size (mm) was estimated by measuring the longest diameter of the tumor. Care of animals and all experimental procedures were approved by the Animal Ethics Committee of Tianjin Medical University Cancer Hospital.

### Statistical analysis

The results are expressed as mean±SD. The Mann-Whitney U test was performed to compare the difference of RNF180 mRNA expression among various cell lines. The difference in tumor growth rate between the two groups of nude mice was determined by repeated-measures analysis of variance. Significance was defined as P < 0.05. All statistical analyses were performed with SPSS 18.0 software.

### Ethics

This study was approved by the Tianjin Medical University Cancer Hospital institutional review board.
